# A New Application of Unsupervised Learning to Nighttime Sea Fog Detection

**DOI:** 10.1007/s13143-018-0050-y

**Published:** 2018-09-20

**Authors:** Daegeun Shin, Jae-Hwan Kim

**Affiliations:** Department of Atmospheric Sciences, Division of Earth Environmental System, Pusan National University, Busandaehak-ro 63beon-gil 2, Geumjeong-gu, Busan, 46241 South Korea

**Keywords:** Sea fog detection, COMS, CALIPSO, Unsupervised learning, EM algorithm

## Abstract

This paper presents a nighttime sea fog detection algorithm incorporating unsupervised learning technique. The algorithm is based on data sets that combine brightness temperatures from the 3.7 μm and 10.8 μm channels of the meteorological imager (MI) onboard the Communication, Ocean and Meteorological Satellite (COMS), with sea surface temperature from the Operational Sea Surface Temperature and Sea Ice Analysis (OSTIA). Previous algorithms generally employed threshold values including the brightness temperature difference between the near infrared and infrared. The threshold values were previously determined from climatological analysis or model simulation. Although this method using predetermined thresholds is very simple and effective in detecting low cloud, it has difficulty in distinguishing fog from stratus because they share similar characteristics of particle size and altitude. In order to improve this, the unsupervised learning approach, which allows a more effective interpretation from the insufficient information, has been utilized. The unsupervised learning method employed in this paper is the expectation–maximization (EM) algorithm that is widely used in incomplete data problems. It identifies distinguishing features of the data by organizing and optimizing the data. This allows for the application of optimal threshold values for fog detection by considering the characteristics of a specific domain. The algorithm has been evaluated using the Cloud-Aerosol Lidar with Orthogonal Polarization (CALIOP) vertical profile products, which showed promising results within a local domain with probability of detection (POD) of 0.753 and critical success index (CSI) of 0.477, respectively.

## Introduction

Fog consists of suspended droplets or ice crystals that reduce visibility to less than 1 km parallel to the surface near the ground (Gultepe et al. 2007). The formation of fog contributes to numerous traffic accidents and delays caused by low visibility (Ahn et al. 2003). Furthermore, the loss of life and damage to property caused by fog are comparable to those caused by tornados and hurricanes (Whiffen 2001; Gultepe et al. 2007, 2009), highlighting the significance of fog monitoring.

Fog develops over both land and sea. In Korea, sea fog is a crucial issue because the Korean peninsula is surrounded by sea on three sides. Although numerous ground observations have been conducted to reduce the losses due to sea fog, it is difficult to understand the overall distribution of sea fog because observation sites are limited to coastlines and islands (Cermak and Bendix 2007, 2008). The use of satellite measurements helps to overcome the temporal and spatial limitations of ground measurements (Ahn et al. 2003). Geostationary satellites in particular have great potential for monitoring the development of weather phenomena as they continually observe the same area with spatial resolution of a few kilometers, with coverage of one quarter of the Earth’s surface area. Due to the highly reflective and homogenous characteristics of the surface of fog, a visible (VIS) channel with high resolution is very effective in distinguishing fog from others. However, during nighttime, only infrared channels are available. This has led to the primary use of the brightness temperature difference (BTD) between the shortwave infrared (SWIR) and infrared (IR) channels to identify fog (Hunt 1973; Eyre et al. 1984). Because this method is simple and highly effective for fog detection, it has been widely applied to polar and geostationary satellites (d’Entremont 1986; d’Entremont and Thomason 1987; Saunders and Kriebel 1988; Bendix and Bachmann 1991; Ellrod 1995; Lee et al. 1997; Park et al. 1997; Bendix et al. 2003; Cermak et al. 2004; Gao et al. 2009; Lee et al. 2011). This method, however, cannot differentiate between fog and stratus because they have similar particle size and altitude.

To separate fog from stratus effectively, Ellrod and Gultepe (2007) proposed an additional threshold combining BTD observed from satellite instrument, with shelter temperatures from surface observing sites. The temperature difference between the infrared cloud surface and the ground is relatively small due to the fact that fog occurs right above the surface of the sea and land. With this approach, Park and Kim (2012) separated sea fog from other clouds by using the difference between infrared cloud top temperature and sea surface temperature (SST), obtained from the Multi-functional Transport Satellite-1 Replacement (MTSAT-1R) and the Advanced Microwave Sounding Radiometer for EOS (AMSR-E), respectively. Similarly, Zhang and Yi (2013) suggested a monthly-dependent dynamic threshold algorithm combining real time brightness temperature (BT) from the Moderate Resolution Imaging Spectroradiometer (MODIS) IR channel (11 μm) with climatological monthly SSTs. Heo et al. (2008) presented a combined method, which includes the BTD, wind speed from the Quick Scatterometer (QuikSCAT), and Laplacian computation. Calvert and Pavolonis (2010) utilized the 3.9 μm pseudo-emissivity, in place of the 3.9–11 μm BTD, which is less sensitive to the scene temperature. Despite these efforts, the accuracy of nighttime fog detection remains low because considerable areas of clear sky and other types of cloud are classified as fog.

Most existing fog detection algorithms are based on supervised learning techniques, which apply predetermined fixed threshold values. It is difficult to adapt these thresholds to take account of continuously changing atmospheric composition, particularly water vapor or aerosol, and these lead to uncertainties in fog detection. To overcome this, we have suggested a new approach that uses threshold values, which are not fixed but variable according to the atmospheric condition of target area, determined from unsupervised learning technique. The Expectation Maximization (EM) method, which is one of the most common method for data mining from incomplete data (Dempster et al. 1977; Zhang et al. 2003; McLachlan and Peel 2000), has been adopted to perform the unsupervised learning. The detailed method will be discussed in Section 4.

There have been some attempts to apply unsupervised learning methods to satellite remote sensing data (Pankiewicz 1995; Papin et al. 2002; Li et al. 2012). However, the limited number of satellite channels and the complex structure of clouds made it difficult to obtain significant performance. Our research, which focuses on the separation between fog and other clouds, uses a different approach to obtain better results.

To apply the unsupervised technique, two independent combined data sets (the spaces used for pattern analysis) were constructed by combining the BT from the Meteorological Imager (MI) onboard the Communication, Ocean and Meteorological Satellite (COMS), with SST from Operational Sea Surface Temperature and Sea Ice Analysis (OSTIA). The unsupervised analysis using the EM algorithm is conducted on each combined data set separately. The retrieved fog pixels were evaluated using data from the Cloud-Aerosol Lidar and Infrared Pathfinder Satellite Observations (CALIPSO) satellite. In this study only nighttime sea fog is considered, for which ground observations and the VIS channel cannot be used.

This paper is organized as follows. Section 2 describes the data sets used. Section 3 examines the characteristics of the combined data sets that are employed for the unsupervised learning. Section 4 explains the method and procedure of our algorithm, and section 5 describes the case studies. Section 6 provides the validation results using CALIPSO measurements. Finally, a discussion and conclusions can be found in section 7.

## Data

COMS is a geostationary satellite that constantly monitors the same area around the Korean peninsula, producing images at 15 min intervals. It carries a MI with four IR channels (3.7, 6.7, 10.8, 12.0 μm) along with the VIS channel (0.67 μm). The spatial resolution is 4 km for the IR channels and 1 km for the VIS channel. In this study, BTs from the SWIR (3.7 μm) and IR (10.8 μm) channels were mainly used for the detection of fog area.

OSTIA is a global high-resolution (~5 km) reanalysis SST data set from the UK Met Office that assimilates various ground observations and satellite data (Stark et al. 2007). It is provided daily and has a root mean square error of 0.39 °C (Stark et al. 2007; Xie et al. 2008; Cha et al. 2011).

The Cloud-Aerosol Lidar with Orthogonal Polarization (CALIOP) onboard CALIPSO was used to validate and identify fog pixels. CALIPSO is a polar orbiting satellite with a revisit time of 16 days. CALIOP is an active lidar sensor designed to acquire vertical profiles of elastic backscatter at two wavelengths (1064 nm and 532 nm) from a near-nadir viewing geometry, and provides vertical profiles of aerosols and clouds. In this study, we mainly used the CALIOP Level 2 vertical feature mask (VFM) product (version 3) that provides vertical information regarding cloud phase or feature type. Additionally, Level 1 attenuated backscatter products were used to identify the feature of each pixel (such as fog, clear sky, and clouds). Kim et al. (2008) showed that the top and base heights of cloud layers estimated from the spaceborne CALIOP and ground based lidar are generally in agreement within 0.10 km.

OSTIA SST and CALIOP profile data were collocated to the nearest MI-COMS pixel. For validation and analysis, the collocated pixels were classified into five categories by means of the CALIOP Level 2 VFM product and Level 1 attenuated backscatter product: “clear”, “fog”, “single-layer cloud”, “multi-layer cloud”, and “totally attenuated”. Totally attenuated refers to a set of pixels that have a profile that is totally attenuated by thick clouds above the sea surface. The classification method for fog is that used by Wu et al. (2015). It examines the CALIOP VFM products, and classifies any cloud layer which has a base attached to the sea surface (allowing 2 bins of the CALIOP vertical resolution above the sea surface, 30 m per bin for low altitudes between −0.5 and 8.2 km) as sea fog. Additionally, pixels with CALIOP VFM surface/subsurface higher than zero (allowing 2 bins above the sea surface) and attenuated backscatter greater than a threshold (0.03 *km*^−1^ *sr*^−1^) are also classified as sea fog. Similarly, other categories are defined by examining cloud profiles obtained from the CALIOP Level 2 VFM product.

## Combined Data Sets for Fog Detection

In satellite images, cloudy and clear regions are characterized by various features (the size, shape and intensity of the regions). In a single channel image, however, fog is difficult to distinguish from other types. For this reason, combined data sets that effectively represent the fog are constructed by combining different types of images. The combined data sets include the brightness temperature difference between SWIR and IR channels (hereafter BTD only refers to the brightness temperature between SWIR and IR channels), and the surface temperature difference between the cloud top and the sea surface located underneath the cloud (STD).

### Brightness Temperature Difference Between SWIR and IR Channels

The BTD commonly used for fog detection is based on the emissivity difference of two IR channels with respect to water droplets. The emissivity of water droplets in the SWIR channel ranges from 0.8 to 0.9, while it is close to 1.0 in the IR channel (Hunt 1973). This results in negative BTD values for fog that mainly consists of water droplets, while BTD values are nearly zero in cloud-free areas. This can be utilized effectively in extracting fog from cloud-free and higher cloud areas. However, BTD values are not always consistent because they depend not only on cloud properties, but also on other factors such as atmospheric composition, surface emissivity, solar zenith angle (only in daytime) and the spatial resolution of the satellite sensor (Ellrod 1995; Bendix et al. 2003, 2004; Cermak and Bendix 2007; Schreiner et al. 2007). Water vapor makes it particularly difficult to identify fog as it contaminates the observed signal; while water vapor makes the BTD biased in the positive direction depending on its amount (Lee et al. 1997), the distribution of water vapor can change greatly with time and space and it is also difficult to measure from limited satellite channels. This can greatly affect fog detection, owing to the quantitatively minor difference of BTD between fog and clear sky features. Furthermore, because stratus have nearly the same BTD as fog, BTD cannot adequately discriminate fog from stratus.

### Surface Temperature Difference Between Cloud Top and Sea Surface Located underneath the Cloud

Sea fog occurs more widely and persists for longer than land fog due to several factors such as its formation process, the presence of sea-salt particles and water vapor. Since the sea has homogeneous surface and small diurnal temperature variation (Gentemann et al. 2003; Kawai and Wada 2007), daily OSTIA ocean temperature can be used to infer sea surface temperature under cloud. The STD is derived by subtracting BT10.8 from SST, and mainly depends on cloud altitude. Although both STD and cloud top height have uncertainties in temperature profile, STD is obtained at all pixels of sea area while cloud top height is retrieved at the pixel where cloud exists.

**Fig. 1:**
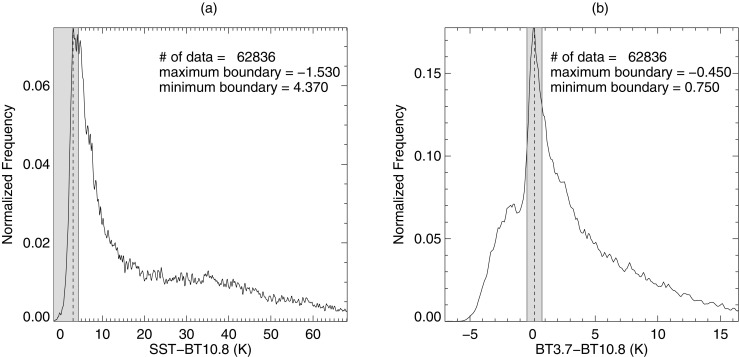
Distribution of (**a**) SST–BT10.8 and (**b**) BT3.7–BT10.8 used to select confident clear pixels in a domain around the Korean peninsula during May to July 2011–2014. The shaded areas correspond to 10% of the total area

For the calculation of STD, reanalysis SST data were employed as an alternative to the BT measurement of sea surface located underneath the cloud. When deriving STD, reanalysis SST should be adjusted towards the BT of the sea surface, since reanalysis SST and satellite observed BT are derived in different ways. The adjustment of SST is carried out via the use of clear pixels that refer to the same area of sea surface in both data sets. Clear pixels are obtained from the distribution of BTD and the distribution of STD (using SST before adjustment). In general, clear pixels, unlike clouds with varying altitude, make up the largest proportion of all the pixels due to their homogeneous surface. As a result, the clear pixels are concentrated around the peak of BTD and STD distributions of climatological data. Therefore we selected pixels around the peak of distribution corresponding to 10% (the value was selected for filtering out non-clear pixels strictly) of the total area, as clear pixels (Fig. 1). In addition, a minimum temperature of 0 °C, an acceptable value for the sea surface around Korean peninsula, was applied to both BT and SST. The pixels satisfying these two conditions were defined as clear pixels for deriving adjusted SST. Verification of the clear pixels obtained in this way was performed using CALIOP profile data, which were found to be more effective in extracting clear pixels than the Level 2 processed MI-COMS clear masking product (showing that ~80% of the clear pixels obtained using our method corresponds to CALIOP clear pixels while ~60% for MI-COMS Level 2 product). The adjusted SST is derived from linear regression between SST and BT10.8 for the clear pixels, and the STD is obtained by subtracting BT10.8 from the adjusted SST.

**Fig. 2:**
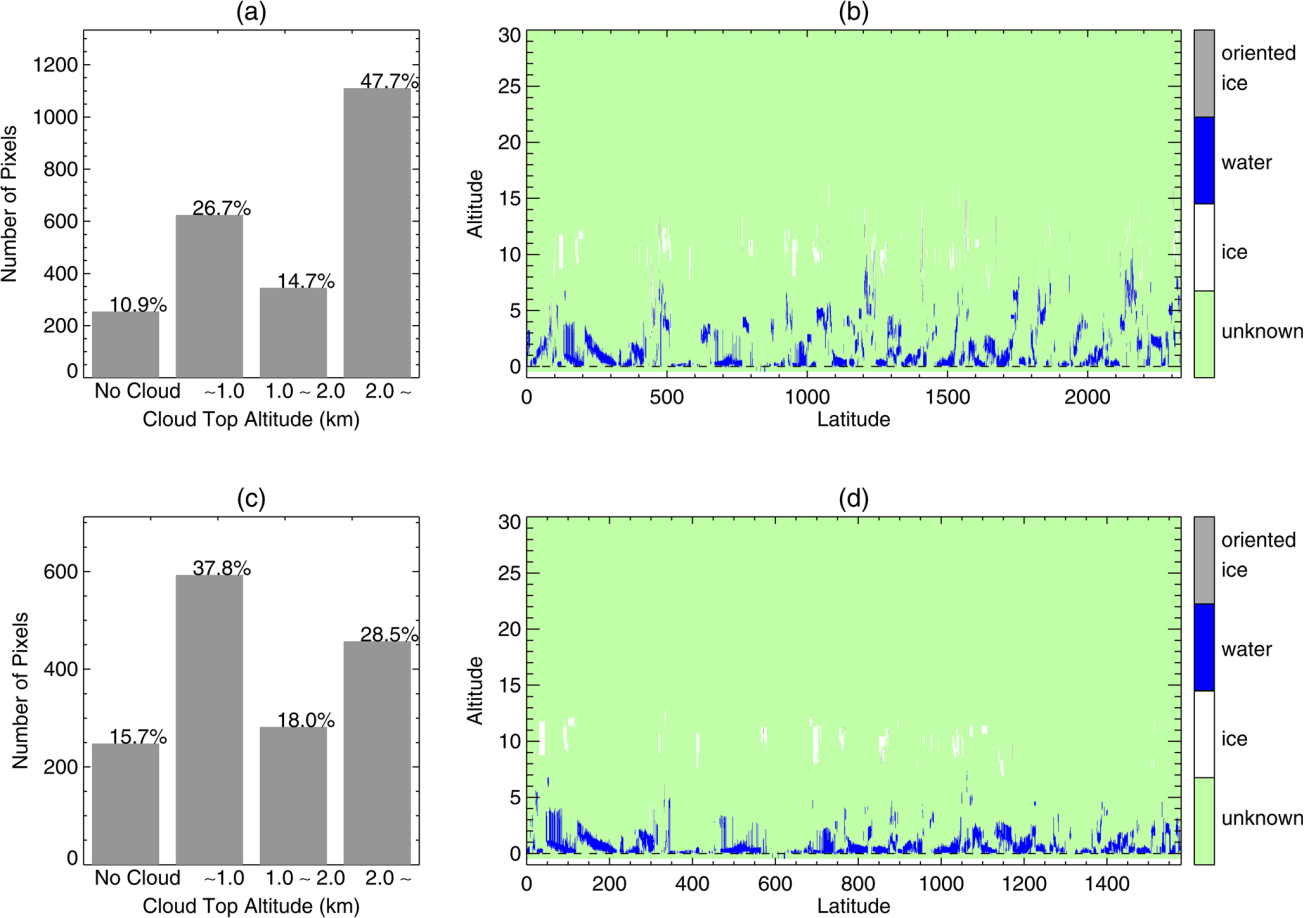
(**a**), (**c**) Cloud altitude distribution of selected fog pixels around the Korean peninsula during May to July 2013. (**b**), (**d**) Accumulated CALIOP ice/water phase vertical profiles of the fog pixels investigated by CALIOP data. Only BTD threshold values are used in (**a**) and (**b**), whereas both BTD and STD threshold values are used in (**c**) and (**d**)

Figure 2 displays the feasibility of using STD for fog detection. The upper panels represent the altitude distribution of pixels selected using the BTD threshold value for fog detection, while the bottom panels use both BTD and STD threshold values. The threshold values of BTD and STD used were − 1.1 and 6.5 K, respectively, derived from climatological data analysis. In the upper panels (BTD only) 47.7% of the pixels selected as fog on the basis of the BTD threshold value are clouds located above 2 km. When the STD threshold is included, the proportion of higher clouds was reduced to 28.5%. This is because the BTD threshold value only discriminates on the basis of cloud properties such as particle size while the STD threshold provides another criterion depending on the altitude of the cloud. However, uncertainties resulting from the variation of temperature profiles and the merging process of two different types of data should also be considered when using STD, as they might result in some discrepancy in STD between clouds at the same altitude but located in different areas. The unsupervised technique described in section 4 suggests a method for minimizing the uncertainties of BTD and STD.

### Climatological Analysis of BTD and STD

**Fig. 3:**
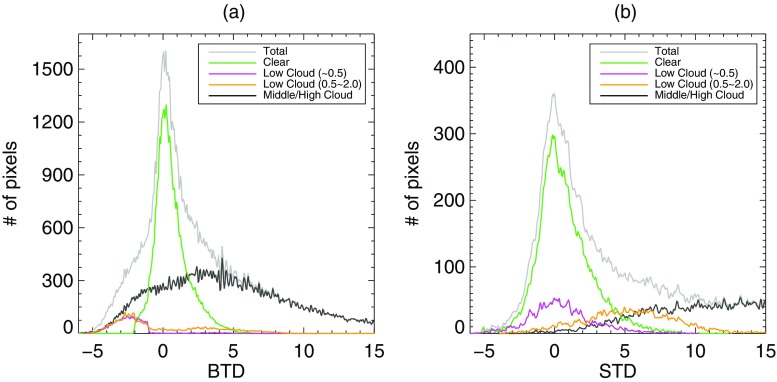
The pixel distribution classified by clear, low cloud and middle/high cloud for (**a**) BTD and (**b**) STD in a domain around the Korean peninsula during May to July of 2011–2014. Clear, low cloud below 0.5 km, low cloud over 0.5 km and middle/high cloud are depicted as green, violet, orange and black lines respectively

Clear sky, low cloud (including fog and stratus) and middle/high cloud have distinguishing features in BTD and STD distribution. Figure 3 demonstrates the characteristics of each scene classified by altitude obtained from MI-COMS cloud top height product. In the BTD distribution (Fig. 3a), low cloud modes are located in the negative area where BTD is less than 0, which corresponds to the aforementioned characteristic of low cloud (including fog and stratus). Clear mode is concentrated around 0 with high density. Ideally, clear scene should be near 0, but it shows slightly positively biased because of water vapor absorption by 10.8 μm (Lee et al. 1997). On the other hand, middle and high clouds are distributed over a broad range of BTD. It is clearly seen that middle and high cloud scene cannot be separated from other features in BTD distribution due to their overlapped distribution range. In contrast, the STD distributions of each scene show a different pattern from BTD distribution (Fig. 3b). The middle and high cloud located at a higher value of STD while clear mode stays around 0 as in the BTD distribution. Furthermore, low cloud below 0.5 km and that above 0.5 km are divided into a mode with lower STD around 0 and a mode with higher STD. In other words, low cloud below 0.5 km has almost same location with clear mode while low cloud above 0.5 km has farther position from clear scene. As a result, low cloud with comparatively higher altitude can be identified in STD distribution. In addition, higher clouds tend to have larger standard deviations due to widely ranging altitudes. These characteristics are utilized to identify modes of each scene.

## Method

**Fig. 4:**
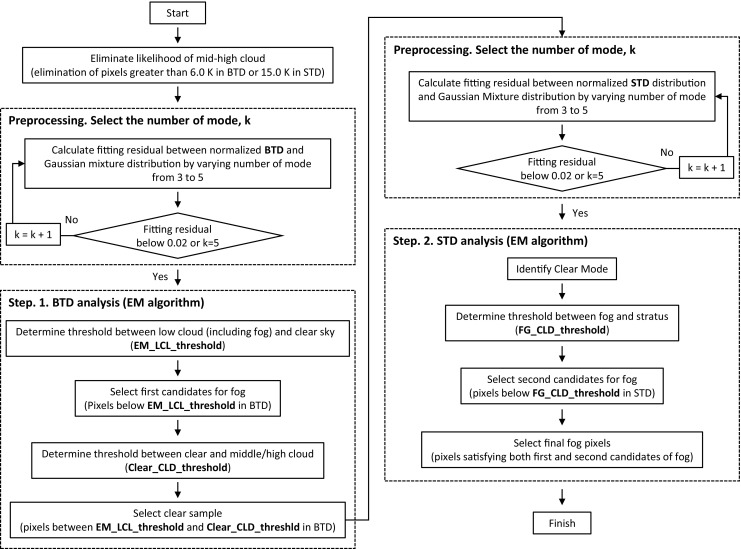
Overview of the sea fog detection algorithm based on the EM-GMM technique

As mentioned above, traditional methods for fog detection using predetermined thresholds are inadequate when atmospheric conditions vary. Our algorithm, using an unsupervised learning method, allows for a more flexible approach in such varying environments, as it analyzes the characteristics of the scene. Figure 4 shows the overview of our algorithm. In this section, a detailed explanation of each process is presented after the description of the EM algorithm.

### EM Algorithm for Gaussian Mixture Model

The EM algorithm searches for the optimal Gaussian mixture model (GMM) through an iteration process (Zhang et al. 2003; McLachlan and Peel 2000). In this study, it was used to find the optimized thresholds as a function of the target domain. This algorithm structuralizes data obtained from the target domain, and represents the data with a GMM. The GMM enables a more practical description of data by reducing the limitation of a unimodal Gaussian distribution. It characterizes data as a combination of several Gaussian models. A Gaussian model centered at mean *μ* with covariance *Σ* and dimension *D* can be written as:

1 $$ \mathrm{N}\left(\left.x\right|\upmu, \Sigma \right)=\frac{1}{{\left(2\pi \right)}^{D/2}\det {\left(\Sigma \right)}^{1/2}}\times \exp \left\{-\frac{1}{2}{\left(x-\mu \right)}^T{\varSigma}^{-1}\left(x-\mu \right)\right\}. $$

Each Gaussian component has its own weighting (*w*_*m*_) satisfying Eq. (2):

2 $$ \sum \limits_{m=1}^M{w}_m=1. $$

The GMM is composed of the weighted sum of normal distributions, which allows the GMM to be treated as a probability density function identical to a unimodal normal distribution. When the parameters of each component are written as θ_*m*_ = {*μ*_*m*_, Σ_*m*_}, the GMM with M components can be expressed as:

3 $$ p\left.\Big(x\right|\Theta \Big)=\sum \limits_{m=1}^MN\left(\left.x\right|{\theta}_m\right){w}_m, $$

where the parameter, Θ = {*θ*_1_, *θ*_2_, …, *θ*_*M*_, *w*_1_, *w*_2_, …, *w*_*M*_}.

The EM algorithm is a technique that finds the maximum likelihood estimate, and consists of the E-Step (Expectation) and M-Step (Maximization). In E-step, the posterior probabilities of each mode with respect to *X* = {*x*_1_, *x*_2_, …, *x*_*k*_} are derived from the given Gaussian parameter (Θ) as described in Eq. (4):

4 $$ P\left(\left.m\right|{x}_k;{\Theta}^{(t)}\right)=\frac{N\left(\left.{x}_k\right|{\theta_m}^{(t)}\right){w_m}^{(t)}}{\sum_{l=1}^M\mathrm{N}\left(\left.{x}_k\right|{\theta_l}^{(t)}\right){w_l}^{(t)}}, $$

where Θ^(*t*)^ denotes the estimated Gaussian parameter obtained after iteration *t*. The largest Gaussian component at point *x* can be identified on the basis of the posterior probabilities of each mode. At the end of this step, the expected complete data log–likelihood function *Q* = (Θ, Θ^*t*^) to be maximized is calculated as:

5 $$ Q\left(\Theta, {\Theta}^t\right)=\sum \limits_{k=1}^K\sum \limits_{m=1}^M\left\{\log {w}_mp\left(\left.{x}_k\right|{\theta}_m\right)\right\}P\left(\left.m\right|{x}_k;{\Theta}^{(t)}\right). $$

In M-Step, the new estimates, *μ*_*m*_^(*t* + 1)^, Σ_*m*_^(*t* + 1)^, *w*_*m*_^(*t* + 1)^ maximizing the log–likelihood function are computed as:

6 $$ {\mu_m}^{\left(t+1\right)}=\frac{\sum_{k=1}^KP\left(\left.m\right|{x}_k;{\Theta}^{(t)}\right){x}_k}{\sum_{k=1}^KP\left(\left.m\right|{x}_k;{\Theta}^{(t)}\right)}, $$

7 $$ {\Sigma_m}^{\left(t+1\right)}=\frac{\sum_{k=1}^KP\left(\left.m\right|{x}_k;{\Theta}^{(t)}\right){\left({x}_k-{\mu_m}^{\left(t+1\right)}\right)}^T\left({x}_k-{\mu_m}^{\left(t+1\right)}\right)}{\sum_{k=1}^KP\left(\left.m\right|{x}_k;{\Theta}^{(t)}\right)}, $$

8 $$ {w_m}^{\left(t+1\right)}=\frac{1}{K}\sum \limits_{k=1}^KP\left(\left.m\right|{x}_k;{\Theta}^{(t)}\right). $$

The obtained estimates serve as input values for E-Step, and are used to calculate the new expected log-likelihood function. This process is repeated until the log-likelihood function is converged to get maximized. The EM algorithm is considerably sensitive to the initial values of the parameters, since it tends to converge to the local maximum. Therefore, in order to obtain reasonable initial values, *k*-means clustering was employed. This speeds up the convergence of the EM algorithm and avoids convergence at local maxima (Hartigan and Wong 1979; Zhang et al. 2003).

### Preprocessing

The EM algorithm is most effective when the target feature is dominant. For this reason, pixels corresponding to assured high clouds where no fog is possible are ruled out prior to implementing the EM algorithm. The elimination of high clouds is carried out by applying threshold values of BTD and STD. Any pixels outside the threshold values are regarded as assured high cloud pixels based on the fact that high cloud shows large positive values in both BTD and STD. The positive values of high clouds are caused by the radiative properties of cirrus clouds for the BTD (Turk and Miller 2005), and the altitudes of high clouds for the STD. The threshold values should be set to contain sufficient number of clear and low cloudy pixels to avoid the over-partition of the fog mode. In this study, we used 6.0 and 15.0 K as the threhold of BTD and STD, respectively, to satisfy this condition as much as possible.

**Fig. 5:**
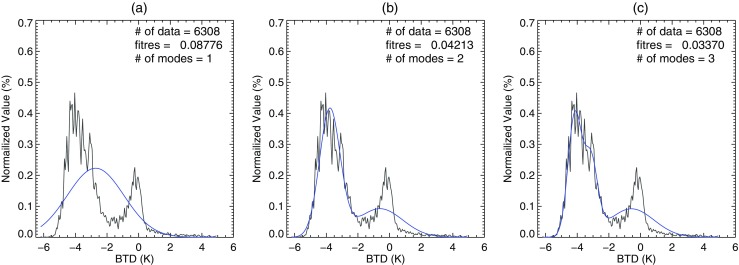
BTD distribution (black) and the Gaussian mixture distribution (blue) obtained from the EM algorithm for increasing numbers of Gaussian modes. The numbers of modes are (**a**) 1, (**b**) 2 and (**c**) 3

To implement the EM algorithm, the number of components should first be selected. As shown in Fig. 5, having more components in the Gaussian mixture distribution leads to a better fit to the original distribution of the original data. However, more components make it difficult to find the modes of the feature as it generates more meaningless components. Therefore, the appropriate selection of the number of modes is required. In our algorithm, the number is determined on the basis of the fitting residual between the Gaussian mixture distribution and the original distribution. To obtain the optimal number of modes, the fitting residual is continuously calculated as the number of modes increases, and the optimal number corresponds to the fitting residual falling below a predetermined value of 0.02. Ideally, it has best performance when the residual value is the smallest. However, in reality, it does not give good results because of overfitting. In this process, the best performance was found when the residual value was 0.02 and the number of modes was between 3 and 5. When the number of modes is overfitted, multiple fog modes can be found by the following conditions in Step 1 and 2. On the other hand, when the number of components is underfitted, it becomes highly difficult to separate the merged features. In order to avoid underestimation, and to maximize calculation efficiency, the numbers are limited between 3 and 5.

### Step 1: Low Cloud Selection from BTD Distribution

The algorithm consists of two steps. The first step is to separate low cloudy (including fog and stratus) from clear pixels using the BTD distribution, and the second is to filter out stratus from fog using the STD distribution. As demonstrated in section 3, a threshold between low cloud and clear sky modes in the Gaussian mixture distribution can be extracted easily in the BTD distribution, while fog and stratus are difficult to separate owing to their similarity in particle size. Conversely, in the STD distribution, it is relatively easy to discriminate fog from stratus based on the altitude difference, while clear and foggy pixels located near or adjacent to ocean surface are hard to separate.

**Fig. 6:**
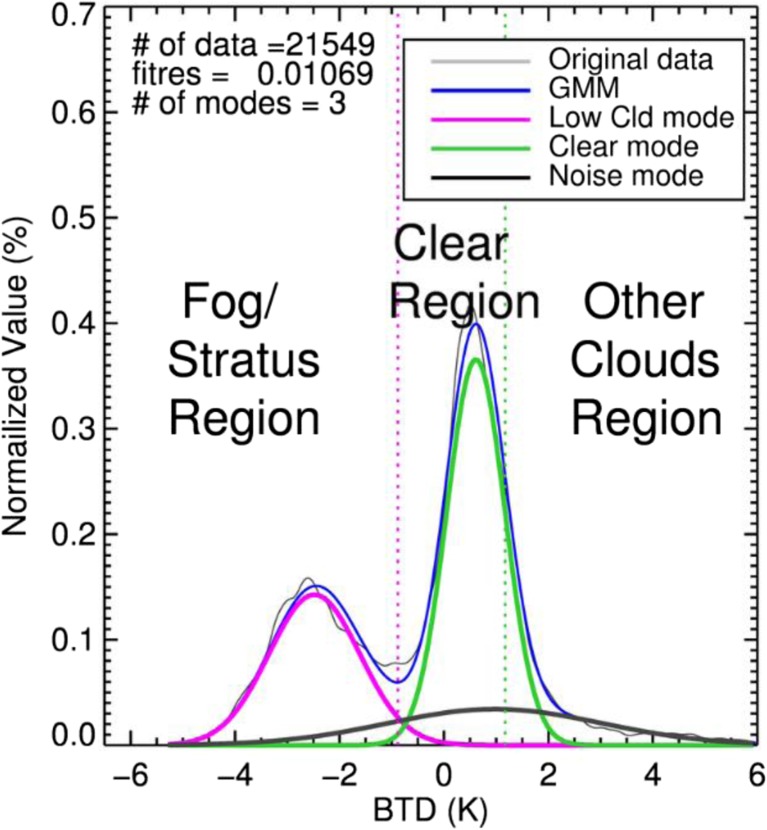
BTD distribution (gray) and the Gaussian mixture distribution (blue) in a domain around the Korean peninsula on 15 June 2012. Low cloud, clear and other cloud (noise) modes are shown by violet, green and black lines, respectively. The vertical dotted lines represent the low cloud threshold derived from the EM algorithm (EM-LCL-threshold, violet), and the threshold between clear and other clouds (Clear-CLD-threshold, green)

The first step focuses on the discrimination of low cloudy from clear pixels. Figure 6 indicates the process of analyzing the BTD distribution. The blue line represents the Gaussian mixture distribution obtained from the EM algorithm while the violet, green and black lines indicate the modes of fog, clear sky, and noise (or other clouds), respectively. Since all Gaussian components are grouped on the basis of unsupervised learning technique without any prior information, a procedure that identifies interesting modes, post processing, is required. The following processes of post processing are constructed on the basis of the characteristics of each features including fog, clear sky and other clouds with regard to BTD and STD as seen in Fig. 3. In step 1, the threshold value between low cloud and clear sky modes (EM-LCL-threshold) is determined along with the assured clear sample that will be used in step 2. These processes can be summarized as follows.

1. Firstly, the EM-LCL-threshold, the local minimum with the largest value of BTD of the Gaussian mixture distribution smaller than zero should be found. Modes located on the negative side of the EM-LCL-threshold are then determined as low cloud mode. Additionally, a local minimum between BTD values of 0 and 1 is used as the EM-LCL-threshold when the BTD value of the center of the Gaussian mode nearest to the local minimum is smaller than the Clim-LCL-threshold (a threshold between low cloud and clear sky modes derived from the climatological BTD distribution). If there is no local minimum that satisfies these conditions, the Clim-LCL-threshold is used instead of EM-LCL-threshold.
2. Clear mode is defined as the nearest Gaussian component to EM-LCL-threshold on the positive side. In order to select assured clear samples, the maximum boundary of clear samples (referred to as the Clear-CLD-threshold) is determined as the sum of the mean and standard deviation of the clear mode. In the analysis of the STD distribution, because it is significant to include assured clear samples along with the smallest amount of high clouds for effective clear mode selection, pixels located in a large STD are excluded from clear sample. Accordingly, pixels between EM-LCL-threshold and Clear-CLD-threshold satisfying STD below 2.5 are chosen as assured clear samples.
3. Pixels with BTD smaller than EM-LCL-threshold are classified as first candidates for fog.

The thresholds determined in this step are shown as dotted lines in Fig. 6. In the next step, stratus pixels are excluded by the analysis of the STD distribution.

### Step 2: Fog Selection from STD Distribution

**Fig. 7:**
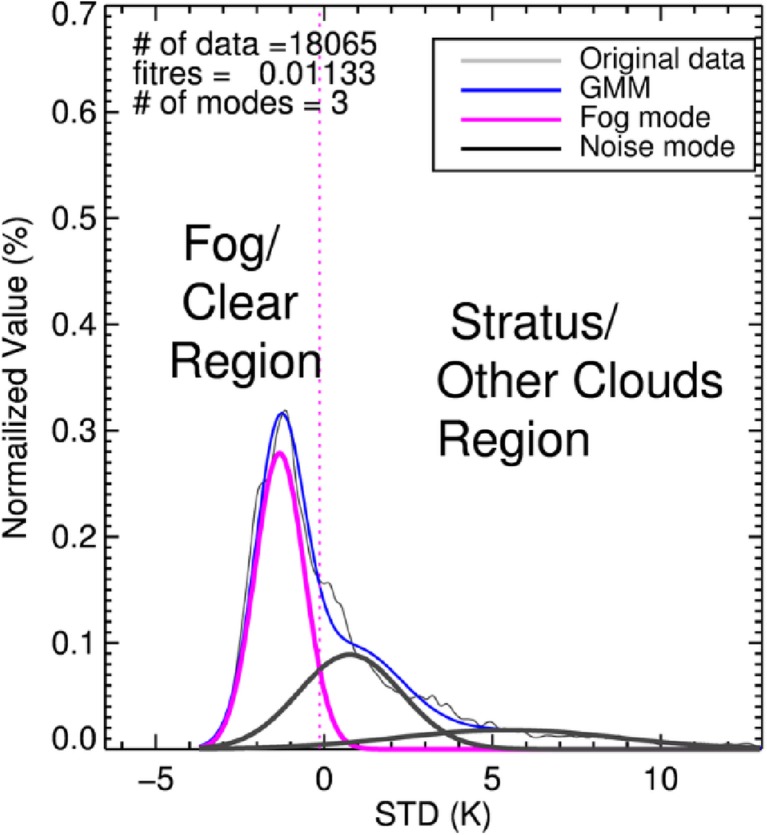
STD distribution (grey) and the Gaussian mixture distribution (blue) for the case in Fig. 6. Fog and other cloud (noise) modes are shown by violet and black lines, respectively. The vertical dotted line represents the threshold between fog and stratus (FG-CLD-threshold) derived from the analysis of the STD distribution

The analysis of the STD distribution is performed only with pixels belonging to the Clear-CLD-threshold in the previous step. They mainly consist of fog, clear sky and stratus samples because most of middle and high clouds have been removed in step 1. In this step, the stratus which could not be separated in step 1 and the remained middle/high clouds are filtered out by analyzing STD distribution. The assured clear samples are used to estimate clear modes, as the STD can vary greatly depending on time and space owing to the uncertainties mentioned in section 3.2. In general, the clear mode has a well-defined Gaussian distribution with small standard deviation around 0 in a given domain and therefore it can be used for a more reasonable identification of fog and stratus. Fog mode is very close to the clear mode, and sometimes form a single mode combined with clear pixels in STD distribution. Therefore fog modes are not distinguished from clear sky modes in STD distribution. Figure 7 shows the process of analyzing the STD distribution. The first mode with the smallest STD and the second mode adjacent to the first one can be seen as clear sky and fog modes, respectively. They can also be interpreted as an over-partitioned clear mode. In step 2, however, it is unnecessary to distinguish between fog and clear sky modes as mentioned above. This step only focuses on the elimination of clouds except fog, which is conducted as follows.

1. Firstly, clear modes are determined as the mode containing the greatest number of the assured clear pixels, or the mode exceeding the assured clear sample ratio of 1/ (the number of mode + 1), or the mode with a negative STD center.
2. Fog modes are identified as a Gaussian component with center located within 2.5 °C of the largest clear sky or fog mode on the positive side. If the clear sky or fog modes have a peak lower than 0.1, that component is regarded as cloud or noise mode. Lastly the mode with the largest value of STD of the clear sky or fog modes is selected as the fog mode from the analysis.
3. The Gaussian component with a center STD value greater than the fog mode is classified as a stratus mode. The point of intersection between the probabilities of the fog and stratus modes is referred to as the FG-CLD-threshold. If the sample size after high cloud elimination is lower than 5% of the original data, the climatological STD threshold value of 6.5 is employed as the FG-CLD-threshold.
4. All pixels that have a smaller STD than the FG-CLD-threshold are classified as second candidates for sea fog. Finally, the pixels that belong to both the first and second candidates for fog are determined as fog.

## Case Studies

### Case 1: 29 June 2011

**Fig. 8:**
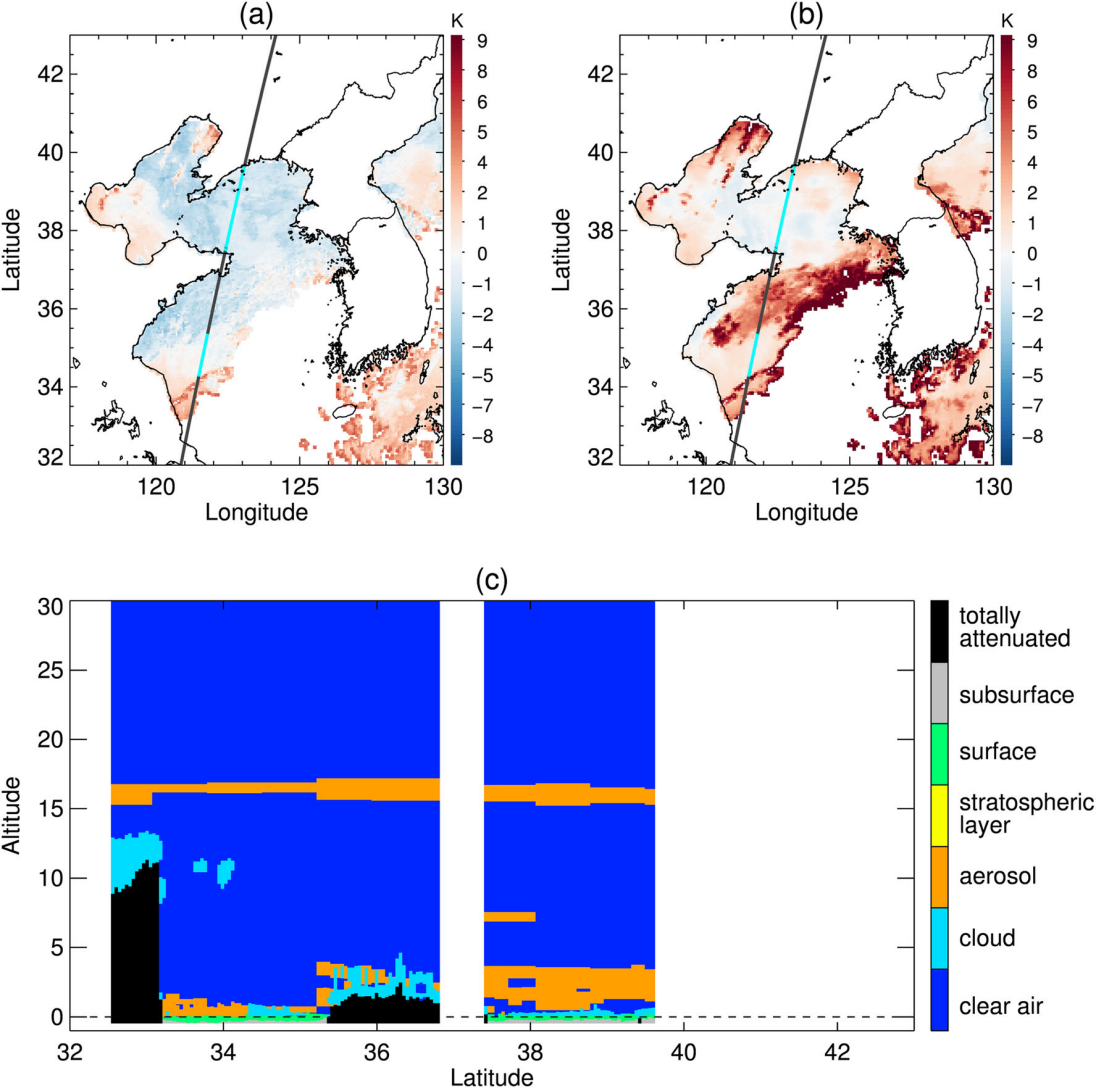
Sea fog event on 29 June 2011. (**a**) BTD and (**b**) STD distributions after eliminating high clouds and land area. The black line is the CALIPSO track and the colored points on the track indicate fog pixels determined from CALIOP vertical profile analysis. **c** CALIOP feature type profile of the track depicted in (**a**) and (**b**) over the sea only

**Fig. 9:**
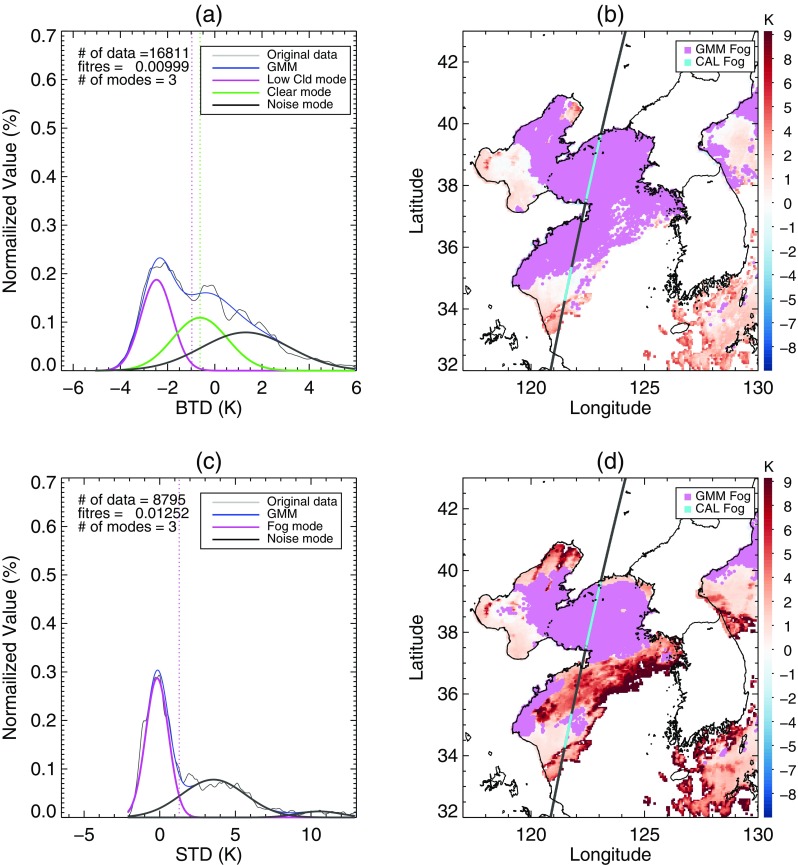
Procedure of BTD and STD analysis for the fog algorithm with EM-GMM applied to the sea fog event on 29 June 2011 shown in Fig. 8. (**a**) and (**b**) are the first step analyzing BTD. (**c**) and (**d**) are the second step analyzing STD. (**a**) and (**c**) are the distribution of BTD and STD that are analyzed as in Figs. 6 and 7, respectively. The pixels identified as low cloud in (**a**) and the pixels identified as fog in (**c**) are shown in violet in (**b**) and (**d**), respectively

**Fig. 10:**
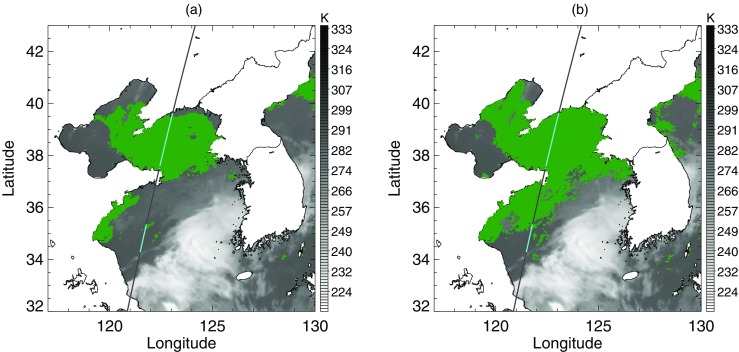
Final fog area detected from an algorithm using (**a**) the EM-GMM technique and (**b**) climatological thresholds of BTD and STD on 29 June 2011

A widespread area of negative BTD is located over the sea west of the Korean peninsula (Fig. 8a). Around the region under the CALIPSO track, Fig. 8b shows that the negative BTD area was divided into two zones by stratus with higher STD. The CALIOP profile verified that the sea fog zones were separated by an area of stratus (Fig. 8c). In the BTD analysis, the upper fog zone around latitude 38°N was included in the first candidates for sea fog, whereas the lower fog zone around latitude 35°N was excluded due to its higher BTD (Fig. 9b). In addition, the stratus crossing the fog zones was not distinguished from fog in the BTD (Fig. 9a, b). The stratus was clearly distinguished in the STD analysis. Figure 9c shows that the threshold between fog and stratus mode was properly identified, which allowed for the elimination of stratus from the final fog area (Fig. 9d). Finally, our method correctly detected the upper fog zone but missed the lower fog zone. Meanwhile, the climatological threshold values of STD and BTD neither eliminated the stratus crossing the fog area nor detected the lower fog stack (Fig. 10).

### Case 2: 24 June 2012

**Fig. 11:**
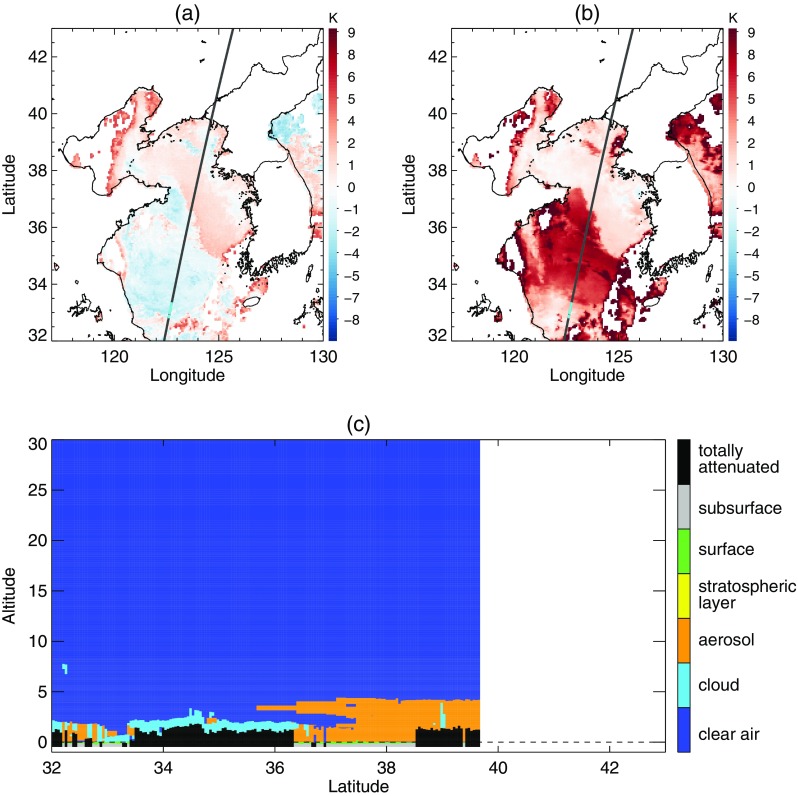
As Fig. 8 but for the sea fog event on 24 June 2012

**Fig. 12:**
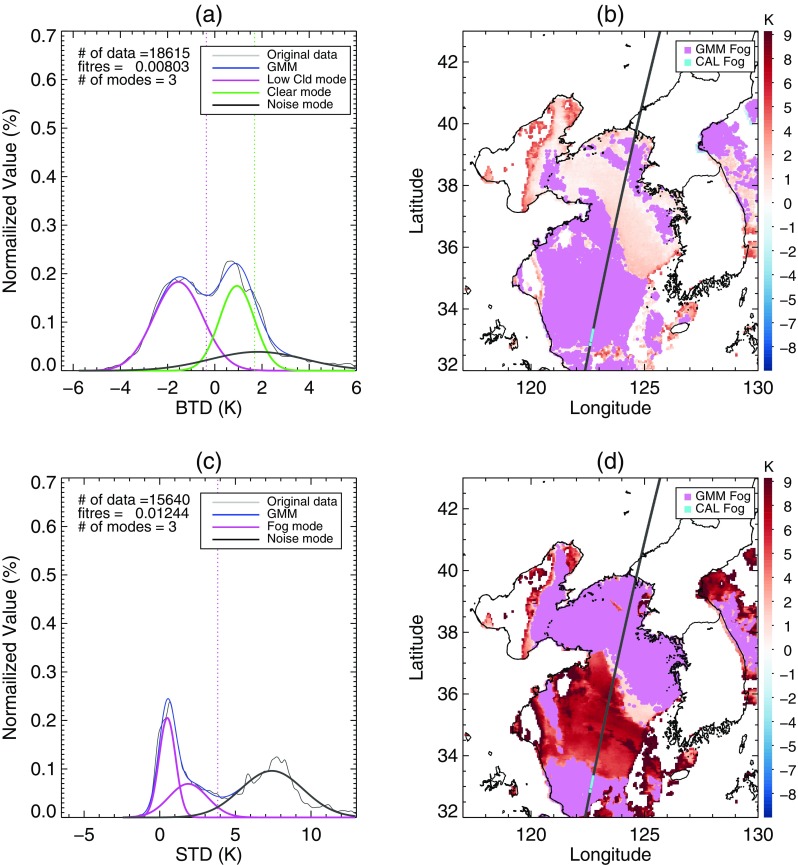
As Fig. 9 but for the sea fog event on 24 June 2012

**Fig. 13:**
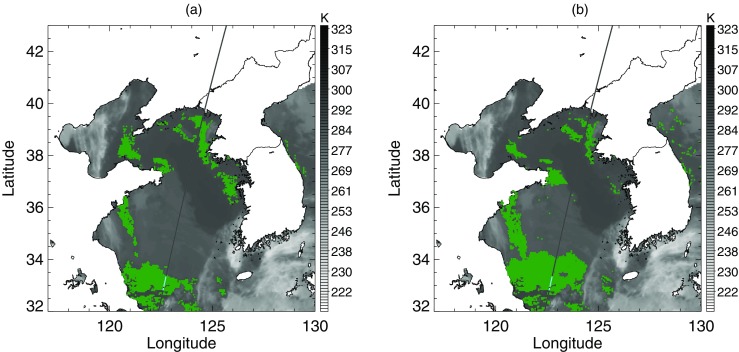
Final fog area detected from an algorithm using (**a**) the EM-GMM technique and (**b**) climatological thresholds of BTD and STD on 24 June 2012

Similar to the situation in Case 1, a widespread area of negative BTD occurred over the sea southeast of the Korean peninsula (Fig. 11a). However, Fig. 11c shows that fog existed only around latitude 31°N in the area of negative BTD. In Fig. 12, the BTD analysis showed two dominant modes for fog and clear sky, and they were properly separated by a threshold derived from the algorithm with EM-GMM technique. In addition, the STD analysis found dominant modes of fog located at lower STD, which resulted in the effective removal of stratus from the second candidates for sea fog. In contrast, a method using climatological thresholds of BTD and STD overestimated the fog area due to the relatively lower STD of stratus caused by unusual atmospheric conditions or inaccurate data at that time (Fig. 13).

## Validation

**Fig. 14:**
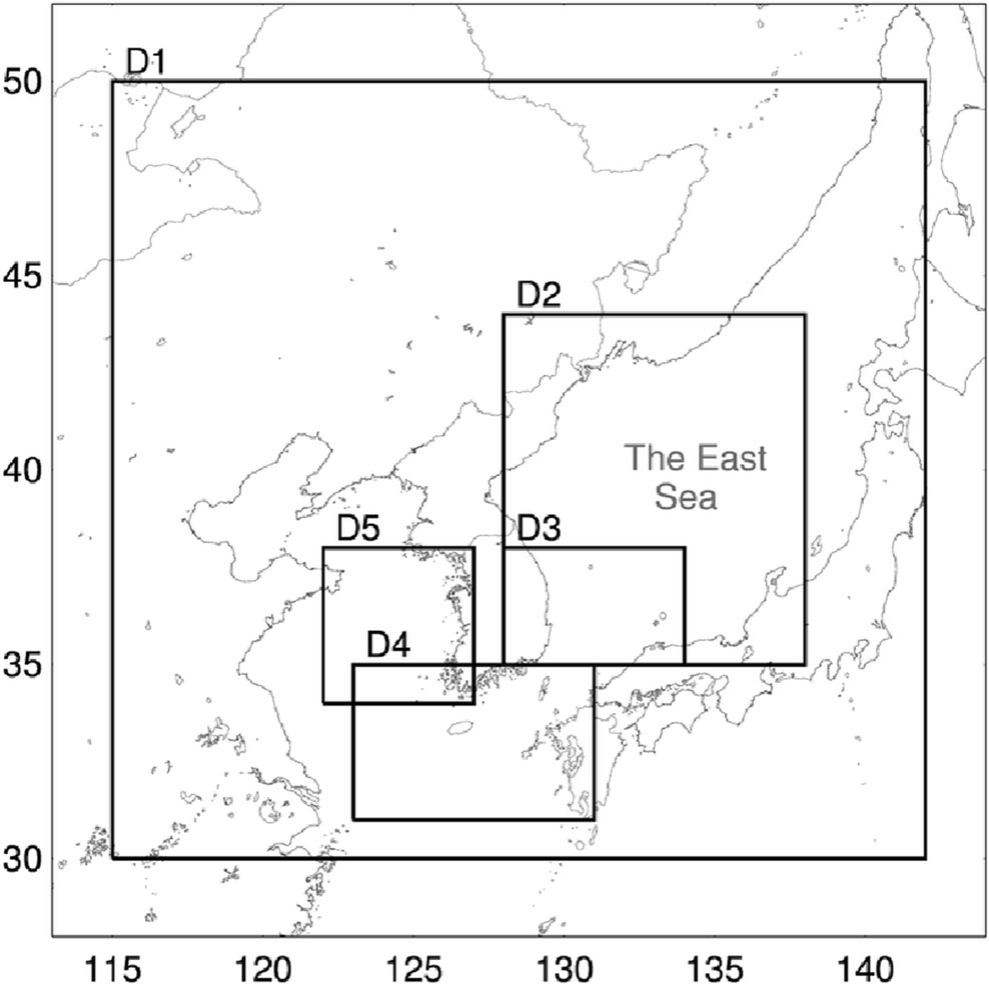
Domains used for validation (D1: [30–50 °N, 115–142 °E], D2: [35–44 N, 128–138 E], D3: [35–38 N, 128–134 E], D4: [31–35 N, 123–131 E], D5: [34–38 N, 122–127 E])

The EM-GMM based fog detection algorithm is validated against the CALIOP profile data during the period of May to July of 2011–2014, when sea fog occurrence is most frequent around the Korean peninsula (Cho et al. 2000). The validation is performed on two regional and three local domains. The two regional domains each cover the seas surrounding the Korean peninsula, and a wide area of the East Sea. The three local domains include parts of the sea surrounding the Korean peninsula (Fig. 14). To quantitatively evaluate the fog detection algorithm, a 2 × 2 contingency table of hits (H), misses (M), false alarms (F), and correct negatives (C) is constructed (Table 1). On the basis of the table, verification statistics including the probability of detection (POD), probability of false detection (POFD), false alarm ratio (FAR) and critical success index (CSI) are calculated as follows (Bendix et al. 2004; Cermak and Bendix 2007, 2011):

9 $$ \mathrm{POD}=\mathrm{H}/\left(\mathrm{H}+\mathrm{M}\right), $$

10 $$ \mathrm{POFD}=\mathrm{F}/\left(\mathrm{F}+\mathrm{C}\right), $$

11 $$ \mathrm{FAR}=\mathrm{F}/\left(\mathrm{H}+\mathrm{F}\right), $$

12 $$ \mathrm{CSI}=\mathrm{H}/\left(\mathrm{H}+\mathrm{F}+\mathrm{M}\right). $$

**Table 1:** Contingency table for fog pixel verification using CALIOP data

	CALIOP Yes	CALIOP No
Algorithm Yes	Hits (H)	False alarms (F)
Algorithm No	Misses (M)	Correct negatives (C)

All these indices range from 0 to 1. Larger values of POD and CSI indicate better detection performance, whereas smaller values are better in POFD and FAR.

For intuitive comparison, the same validation is conducted for two kinds of traditional algorithm. One algorithm, BTD_STD, applies BTD and STD threshold values derived from climatological analysis, after the same preprocessing as in our algorithm. Another algorithm, COMS_THS, uses a set of thresholds (IR1, IR1–IR2, IR1–WV, SWIR–IR1) for MI-COMS (National Institute of Meteorological Research 2009). Our algorithm is referred to below as EM_FOG for convenience. The validation results of the three algorithms for the five domains are summarized in Table 2. Additionally, for a detailed analysis, the fog pixels detected by each algorithm are examined and categorized using CALIOP profile data (Table 3).

**Table 2:** Verification scores of fog detection algorithms by domain

	POD	POFD	FAR	CSI
Domain 1 (D1, Regional)				
BTD_STD	0.701	0.073	0.606	0.337
COMS_THS	0.307	0.027	0.569	0.218
EM_FOG	0.592	0.043	0.517	0.362
Domain 2 (D2, Regional)				
BTD_STD	0.838	0.091	0.557	0.408
COMS_THS	0.387	0.036	0.515	0.274
EM_FOG	0.738	0.055	0.465	0.450
Domain 3 (D3, Local)				
BTD_STD	0.801	0.054	0.603	0.361
COMS_THS	0.367	0.029	0.639	0.223
EM_FOG	0.753	0.026	0.434	0.477
Domain 4 (D4, Local)				
BTD_STD	0.611	0.062	0.679	0.266
COMS_THS	0.245	0.016	0.571	0.185
EM_FOG	0.561	0.028	0.509	0.355
Domain 5 (D5, Local)				
BTD_STD	0.794	0.112	0.650	0.321
COMS_THS	0.392	0.057	0.659	0.223
EM_FOG	0.736	0.081	0.593	0.355

**Table 3:** CALIOP classification of pixels detected as fog by the three algorithms

	Fog	Clear	Cloud	Total
Domain 1 (D1, Regional)				
BTD_STD	2777	311	3967	7055
COMS_THS	1215	37	1565	2817
EM_FOG	2347	285	2232	4864
Domain 2 (D2, Regional)				
BTD_STD	1256	120	1458	2834
COMS_THS	596	10	606	1195
EM_FOG	1105	93	866	2064
Domain 3 (D3, Local)				
BTD_STD	133	15	187	335
COMS_THS	61	2	106	169
EM_FOG	125	9	87	221
Domain 4 (D4, Local)				
BTD_STD	217	37	423	677
COMS_THS	87	4	112	203
EM_FOG	199	21	185	405
Domain 5 (D5, Local)				
BTD_STD	235	24	412	671
COMS_THS	116	7	217	340
EM_FOG	218	21	297	536

In all domains, COMS_THS shows mostly poor POD and CSI as it underestimates fog. It seems that unnecessary or inappropriate thresholds are applied too strictly. Meanwhile, BTD_STD has higher levels of POD along with higher FARs than others (except for Domain 5), because it involves not only fog but also a significant numbers of clear sky and other cloud pixels. Lastly, the EM_FOG algorithm has even lower FAR than BTD_STD as it eliminates more non-fog pixels. On the other hand, the EM_FOG algorithm sometimes filters out unexpected fog pixels in company with the non-fog pixels, which results in lower POD than the BTD_STD algorithm. In domain 1 (D1), the largest domain, the EM_FOG algorithm had significantly fewer hits, corresponding to only ~85% of those obtained with the BTD_STD algorithm. Similarly, in domain 2 (D2), EM_FOG algorithm had fewer hits coupled with lower POD than the BTD_STD algorithm. However, unlike in the regional domains, in the local domains 3, 4, and 5 (D3, D4 and D5), EM_FOG detected almost the same number of fog pixels as the BTD_STD algorithm. Therefore, it has an almost identical level of POD as the BTD_STD algorithm. In addition, CSI and FAR are largely improved in EM_FOG algorithm as seen in the remarkable decrease in the number of non-fog pixels. This is apparent in domain 3, where FAR falls below 0.5 and CSI almost reaches 0.45. In a local domain, the spatial variation of atmospheric conditions is limited. On the contrary, a regional domain is likely to have spatially inconsistent atmospheric conditions, not reflected in analysis data, which can lead to uncertainties in sea fog detection using thresholds, particularly in such a large domain.

## Discussion and Conclusions

**Fig. 15:**
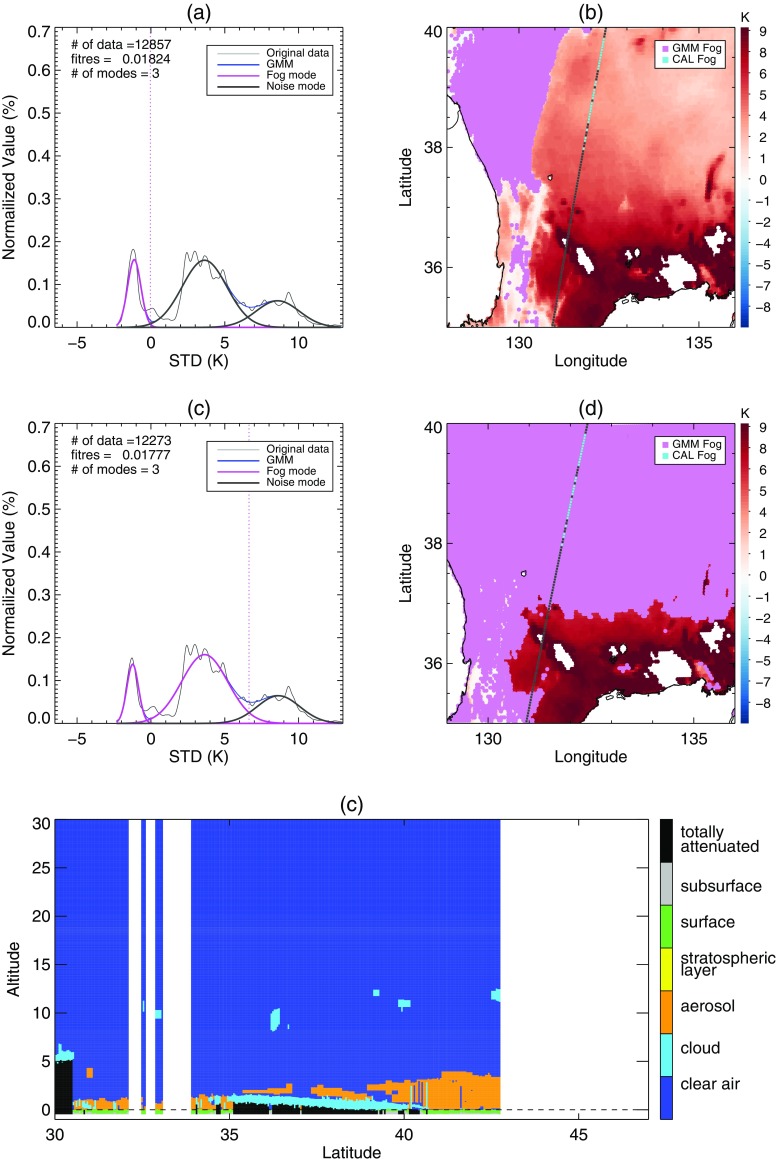
STD distribution analysis (step 2) for 7 July 2012, over the area of latitude 35–40°N and longitude 128–136°E. (**a**) Gaussian components overlapped on the distribution of the original data. (**b**) Latitude–longitude STD distribution showing the fog/clear mode in violet. The black line on the map is a CALIPSO track, and the colored points on the track indicate fog pixels obtained from CALIOP vertical profile analysis. (**c**) and (**d**) is the same with (**a**) and (**b**), respectively, but with slightly different longitude of 129–136° E. (**e**) is CALIOP feature type profiles of the track

As seen in the previous section, results from the EM_FOG algorithm are promising, especially on local domains. However, this method should be used with care for the following reasons. Firstly, in the selection of the optimal domain size, it is still difficult to present a clear criterion. Figure 15 depicts the second step of the EM_FOG algorithm on July 7 2012, but for slightly different domains. In Fig. 15a and b, only the first mode with the lowest central value of STD was selected as the fog/clear sky mode. On the other hand, in Fig. 15c and d, both first and second modes were selected as fog/clear sky modes owing to the increased proportion of clear samples caused by the change of domain. This demonstrates that a small difference in domain can lead to a substantial difference in the final fog pixel selection. Secondly, cases with ambiguous cloud features such as developing clouds can cause some confusion for our algorithm. As seen in Fig. 15e, which depicts CALIOP VFM profiles of the case shown in Fig. 15a and b, the fog is a monolithic stack united with stratus. This kind of fog is difficult to distinguish from stratus because there is no distinct threshold between them. This makes an accurate analysis of distribution difficult. For this reason, the EM_FOG algorithm was not able to detect fog effectively in both Fig. 15a, b and c, d compared with other cases.

**Fig. 16:**
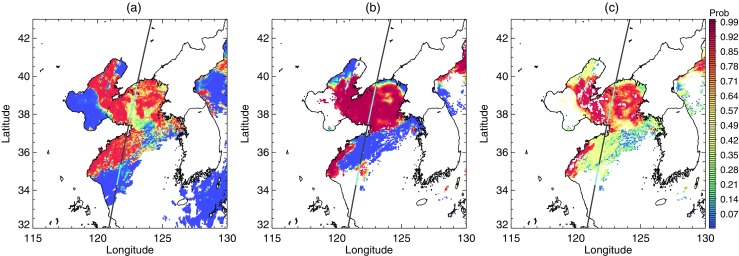
Fog probability derived from EM algorithm for the analysis of (**a**) BTD distribution and (**b**) STD distribution. (**c**) is an overall probability combined (**a**) with (**b**)

These problems occur because the properties of the feature are not adequately reflected in the data. The observed data only contain partial information regarding the feature. The missing information leads to uncertainties in the final results. Since there are no perfect data, it is significant to extract available information as much as possible from incomplete data. In addition, along with the increasing amount of data accompanied by the advance of technology, extraction of the desired information from enormous data mountains has also emerged as a crucial issue. Accordingly, a number of techniques for extracting meaningful information from the data have continuously been developed. This emphasizes the significance of selecting an appropriate technique corresponding the purpose of research. In this context, we have presented a successful application of an unsupervised learning technique to nighttime sea fog detection. Our algorithm can provide not only as the presence of fog, but also the probability of foggy information in a given pixel (Fig. 16). Above all, this study is significantly different from the previous studies with fixed threshold values from supervised learning by utilizing varying threshold values through unsupervised learning, which is appropriately applied to the changing atmospheric condition. Furthermore the presented algorithm has a potential for future application, and which is not confined to fog detection. In particular, in satellite remote sensing applications that employ various threshold values, it is likely that there will be many opportunities for the application of unsupervised techniques. Accordingly, on the basis of our results, it is expected to be continued further researches and challenges.
